# Primary synovial sarcoma of the right heart involving the tricuspid valve in an elderly Chinese woman: a case report

**DOI:** 10.1186/s13000-015-0300-6

**Published:** 2015-06-26

**Authors:** Zhen Huo, Haizhen Lu, Qi Mao, Zhengyu Jin, Huanwen Wu, Xiaoli Feng, Yu Xiao, Yining Wang, Lina Guo

**Affiliations:** Department of Pathology, Peking Union Medical College Hospital, Chinese Academy of Medical Sciences & Peking Union Medical College, No.1Shuaifuyuan, Wangfujing Street, Dongcheng District, Beijing, 100730 China; Department of Pathology, Cancer Hospital/Institute, Chinese Academy of Medical Sciences, Beijing, 100021 China; Department of Cardiac Surgery, Peking Union Medical College Hospital, Chinese Academy of Medical Sciences & Peking Union Medical College, Beijing, 100730 China; Department of Radiology, Peking Union Medical College Hospital, Chinese Academy of Medical Sciences & Peking Union Medical College, Beijing, 100730 China

**Keywords:** Synovial sarcoma, Heart, SS18 rearrangement

## Abstract

Described herein is a 51-year-old woman with abdominal discomfort who was found to have a pericardial effusion and a large mass in her right heart by computed tomography scan and who then underwent tumour resection surgery. The tumour was so extensive that it involved the right atrium, the right ventricle and the tricuspid valve, and encompassed the right coronary artery. The patient had no significant medical history, and no tumour was found at any other site. The morphology of the tumour mimicked carcinosarcoma, exhibiting mixed epithelioid and spindle elements and it was difficult to differentiate the diagnosis even by immunohistochemical stains. Then, the final diagnosis of primary biphasic synovial sarcoma of the heart was established based on the finding of SS18 rearrangement. This is a highly intriguing rare case that may represent a diagnostic pitfall, particularly regarding frozen section. The patient will receive chemotherapy, and we will pursue follow-up.

## Background

Primary cardiac synovial sarcoma (PCSS) was first described in 1978 [1]. PCSS as an entity may present as a biphasic tumour composed of spindled and epithelioid areas or as a monophasic tumour, characterised by the spindle cell component only. To our knowledge, primary cardiac tumours are uncommon, occurring less frequently than metastatic tumours (in a ratio of approximately 1:20 ~ 40) with an incidence in autopsy series ranging only from 0.001-0.03 % [2, 3]. Most of the primary cardiac tumours are benign and approximately 25 % of them are malignant, of which the majority are sarcomas [4]. PCSS accounts for approximately 4.2 % of primary cardiac sarcomas [5], which is extremely rare. We report a case of biphasic PCSS of the heart, which arose from the right atrium and involved the right ventricle and the tricuspid valve.

## Case presentation

**Fig. 1:**
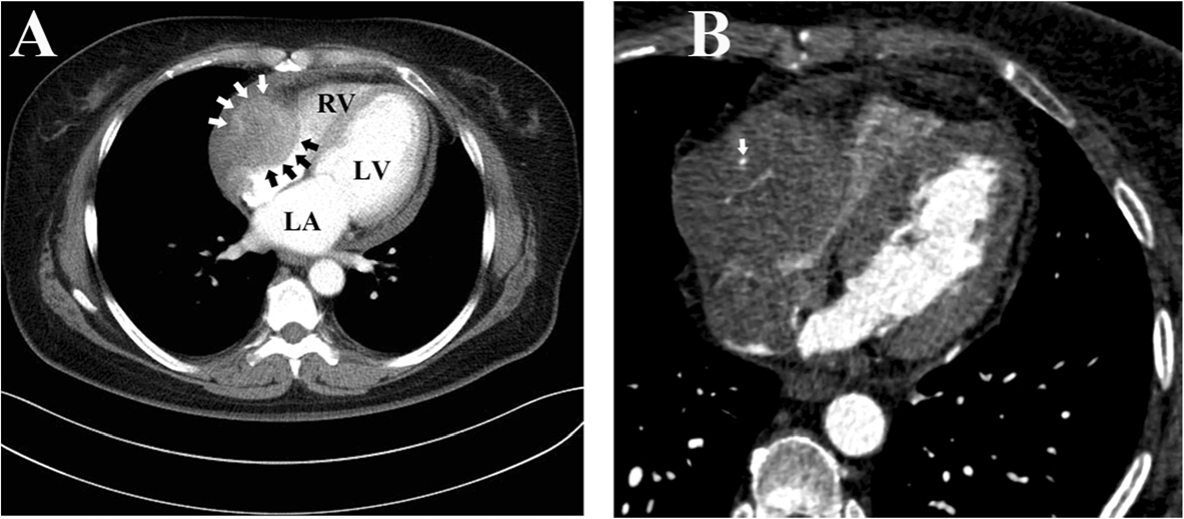
**a**. Computed tomography showing a tumour mass in the right atrium with irregular enhancement and, also a small pericardial effusion (arrow indicates the tumour). **b**. Computed tomography showing the right coronary artery was also involved by the tumour (arrow indicates the coronary artery)

A 51-year-old woman presented with a 1-month history of recurrent abdominal discomfort and a 7-day history of pericardial effusion managed at her local hospital. After the removal of 200 ml of bloody pericardial effusion by pericardiocentesis, she experienced remarkable symptomatic improvement. She went to our outpatient department for follow-up treatment. She had no significant medical history. An electrocardiogram was unremarkable. Echocardiography revealed a heterogeneous mass in her right atrium and a small pericardial effusion. A computed tomography (CT) scan confirmed the findings of a large mass (7.0x8.3 cm) in the right atrium, with irregular enhancement on enhanced CT scan (Fig. 1a) and involvement of a coronary artery as well (Fig. 1b). Coronary arteriography revealed more than 60 % stenosis of the middle segment of the right coronary artery. Computed tomography of the chest and abdomen were unremarkable.

At surgery, the patient was placed on cardiopulmonary bypass using bicaval cannulation through a median sternotomy approach. A large mass was observed arising from the right atrioventricular groove and extending to the anterior of the right atrium. By opening her right atrium, the surgeons observed that the main body of the tumour was located in the wall of the right atrium, involving the right ventricle as well as the anterior leaflets of the tricuspid valve and encompassing the stem of the right coronary artery. The surgeons were diligent in removing the tumour; they also resected some tricuspid valve and involved the right ventricle wall, after which coronary artery bypass grafting was performed.

**Fig. 2:**
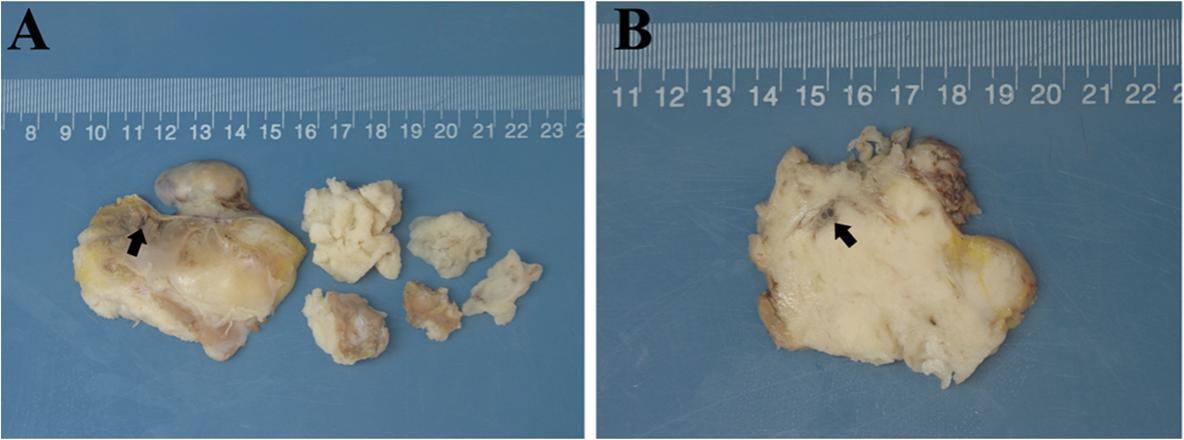
**a**. Gross appearance of the resected tumour showing a main tumour mass and pieces of fragmented tissue, tumour surface with partial encapsulation, and visible cardiac papillary muscle (indicated by the arrow). **b**. The cut surface of the tumour is solid and tan-white in colour with a few haemorrhagic areas and the stem of the right coronary artery is encompassed by the tumour (indicated by the arrow)

**Fig. 3:**
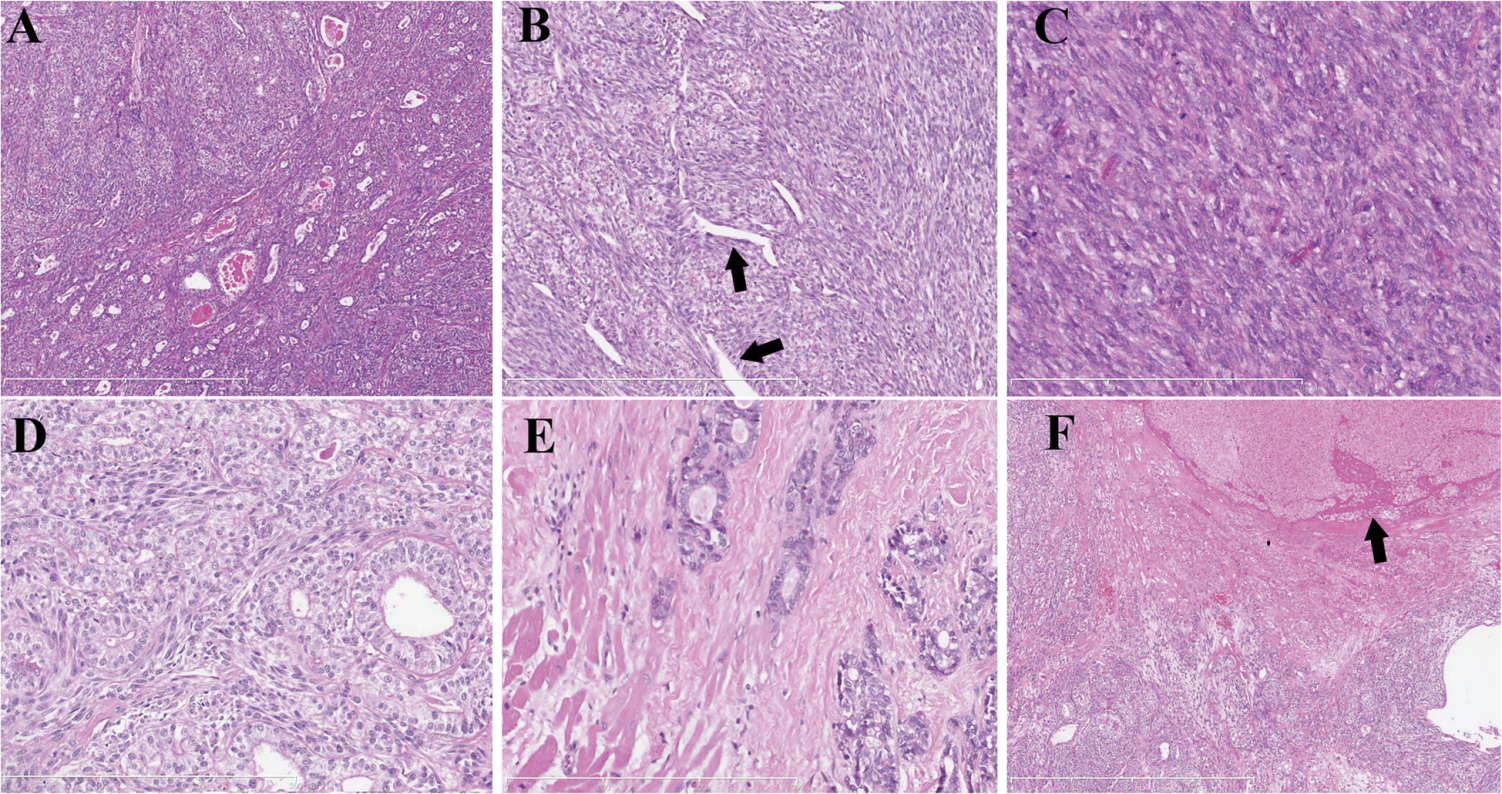
**a**. The tumour consisted of a mixture of spindle cell and epithelioid components with intraluminal eosinophilic material mimicking carcinosarcoma (hematoxylin and eosin stain, 75×). **b**. The focal slit-like structures in some areas (indicated by the arrow, hematoxylin and eosin stain, 150×). **c**. The spindle cells showing eosinophilic cytoplasm and round to oval nuclei with nuclear pleomorphism and with mitoses readily observed (hematoxylin and eosin stain, 300×). **d**. The epithelioid element was the main component in most areas, composed of well-differentiated glandular structures with lighter cytoplasm, admixed with some spindle components (hematoxylin and eosin stain, 300×). **e**. Close to the tumour’s border only glandular structures were present, mimicking adenocarcinoma (hematoxylin and eosin stain, 300×). **f**. The coronary artery (indicated by the arrow) was observed to be encompassed by the tumour, and thrombus was present in the lumen (hematoxylin and eosin stain, 75×)

**Fig. 4:**
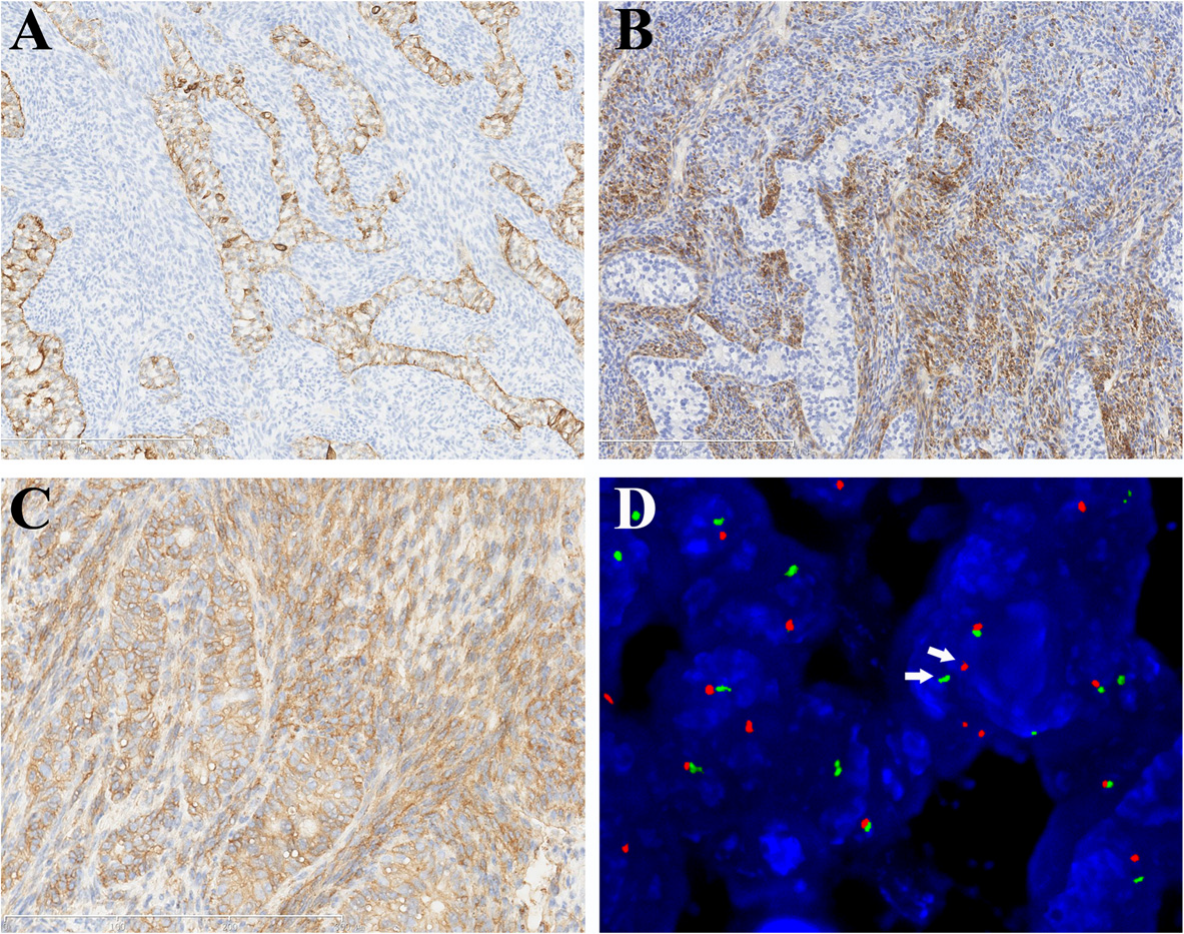
**a**. Epithelioid components are immunopositive for CK19 (150×). **b**. The spindle cells are immunopositive for calponin (150×). **c**. Both epithelioid and spindle tumour cells are immunopositive for CD99 (300×). **d**. FISH demonstrating a balanced rearrangement of the SS18 locus in the majority of tumour cells. The arrow indicates the split signal pattern of red and green signal (1000×)

Gross examination showed the tumour to be generally grey-white tissue with a total size of 8x8x5 cm, with partial surface encapsulation (Fig. 2a); the cut surface was grey-pink, solid and firm with a few haemorrhagic areas (Fig. 2b). The size of the largest tissue fragment was 6.5x5.5x5 cm. Microscopically, the tumour displayed a mixture of spindle cell and epithelioid components in most areas, which mimicked carcinosarcoma (Fig. 3a) with a few slit-like structures (Fig. 3b) and necrosis. Only a small number of tumour areas were composed of pure spindle cell elements, which exhibited eosinophilic cytoplasm and round to oval nuclei with nuclear pleomorphism. Mitoses were readily observed (8-10/10 high-powered fields) (Fig. 3c). The epithelioid element was the main component in most mixed areas, comprising well-differentiated glandular structures with lighter cytoplasm and intraluminal eosinophilic material, and was observed to be blended with less voluminous spindled components (Fig. 3d). In very few areas close to the tumour border, there were solely obvious glandular structures mimicking adenocarcinoma (Fig. 3e). The right coronary artery was involved by the tumour and thrombus could be observed within the lumen (Fig. 3f). A small tumour embolus could be seen in the blood vessel. Immunohistochemical staining showed that the epithelioid components were positive for cytokeratin, CK19 (Fig. 4a) and S-100(focally). The spindle cell components were positive for calponin (Fig. 4b) and vimentin. Both components were positive for BCL-2 and CD99 (Fig. 4c). The Ki-67 index was 25 % in both components. Desmin, WT-1, calretinin and SMA were negative in both components. Details regarding the antibodies used are given in Table 1. Fluorescence in situ hybridization (FISH) showed the rearrangement of SS18 (Vysis SS18 Break Apart FISH Probe Kit, Abbott Molecular Inc., USA) in the tumour cells (Fig. 4d). Based on the above-mentioned findings, the final diagnosis was primary synovial sarcoma of the right heart involving the tricuspid valve. This study was approved by the Ethics Committee of the Peking Union Medical College Hospital, and informed consent was obtained from the patient.

**Table 1:** List of various antibody markers in the present study

Antibody markers	clone	Dilution	Producer
cytokeratin	AE1/AE3	1:200	Dako,Glostrup, Denmark
CK19	RCK108	1:100	Dako,Glostrup, Denmark
S-100	Polyclonal	Prediluted	ZSGB-BIO, Beijing, China
Calponin	EP63	1:100	ZSGB-BIO, Beijing, China
Vimentin	V9	1:200	Dako,Glostrup, Denmark
BCL-2	124	1:50	Dako,Glostrup, Denmark
CD99	12E7	1:100	Dako,Glostrup, Denmark
Ki-67	MIB-1	1:100	Dako,Glostrup, Denmark
Desmin	D33	1:100	Dako,Glostrup, Denmark
SMA	1A4	1:100	Dako,Glostrup, Denmark
Calretinin	Polyclonal	Prediluted	ZSGB-BIO, Beijing, China
WT-1	6 F-H2	1:50	Dako,Glostrup, Denmark

## Conclusions

Synovial sarcoma (SS) is a distinctive entity, approximately 90 % of which arise in the extremities, with a predilection for the regions adjacent to large joints. Occasionally, the tumour may involve unusual sites throughout the body, including the head and neck, heart, pleura, abdomen, kidney, prostate, vulva, etc., which may be difficult to correctly diagnose [6]. Primary cardiac synovial sarcoma (PCSS) occurs infrequently. Wang *et al.* [5] reviewed 54 isolated literature reports before 2012 and found that there were 60 patients with PCSS, and only 16 cases of intra-cardiac biphasic SS. To date, less than 80 patients in total have been reported in the literature [7–17]. Here, we report a patient who was found to have a bloody pericardial effusion and a large right atrium mass who was diagnosed with biphasic PCSS mimicking carcinosarcoma. We arrived at our diagnosis because of the histological characteristics, including immunohistochemical staining and rearrangement of the SS18 gene.

PCSS may occur at any age (from 13 to 70 years), although it more frequently occurs during the fourth decade with a mean age of 37 years, and a male/female ratio of approximately 3:1 [5, 7–17]. The most common symptom is dyspnoea (in 68 % of patients). The patients with PCSS more readily exhibit gastrointestinal and systemic symptoms, and they frequently also have pericardial effusions compared to patients with benign cardiac tumours. The tumour often displays a larger tumour size of more than 5 cm in diameter. The most common location is the pericardium. Other locations include the right atrium, left atrium, tricuspid valve, right ventricle, left ventricle, mitral valve and pulmonary valve sequentially and the tumour readily involves the pericardium [5, 9, 11]. Clinically, this patient was an elderly woman who had abdominal discomfort who was found to have a bloody pericardial effusion by pericardiocentesis, with the finding of a mass that involved the right atrium, the right ventricle and the tricuspid valve.

Macroscopically, the tumour was often polypoid or lobulated with a smooth or well-circumscribed surface and a broad base. Microscopically, Wang *et al.* reviewed 51 reported occurrences with pathological information, among which 27 (52.9 %) were monophasic and 24 (47.1 %) were biphasic PCSS [5]. Histologically, this case has typical biphasic morphology with rich glandular structures in most areas, which may lead to a diagnostic pitfall. It is difficult to discriminate this biphasic PCSS from metastatic carcinosarcoma by the morphologic features alone, particularly at frozen section; moreover, mesothelioma, myoepithelial carcinoma, and malignant peripheral nerve sheath tumour with epithelial differentiation must also be considered in the differential diagnosis (details provided in Table 2). Although the immunohistochemical stains, such as CK19, S-100, BCL-2, CD99, calponin, etc., could aid in making the diagnosis, the role of immunohistochemical staining is limited. Cardiac metastatic carcinosarcoma is particularly difficult to distinguish from SS because the former occur more frequently than primary tumours and also express epithelial markers. However, the patients with metastatic carcinosarcoma tend to be older and often have a history of malignant tumour, and the tumour cells tend to display nuclear pleomorphism. Whereas our patient was previously healthy and the medical examination did not reveal masses in other sites, metastatic carcinosarcoma could not be excluded because of the similar morphology. TLE1 may be a useful marker because it is negative in carcinosarcoma, although TLE1 shows limited specificity in the diagnosis of SS as it is expressed in several neoplasms in the differential diagnosis, particularly those of peripheral nerve sheath origin [18]. Because mesothelioma shares expression of epithelial markers, WT-1 and calretinin, mesothelioma could be excluded because WT-1 and calretinin were negative in the tumour. Myoepithelial carcinoma exhibits tubular and ductal elements resembling biphasic SS, which may be a pitfall, and also often expresses S-100, SMA and epithelial markers; however, it could be excluded because SMA was negative in our case. Although malignant peripheral nerve sheath tumour (MPNST) with epithelial differentiation is rare, and mostly occurs in patients with neurofibromatosis type 1, it must be distinguished in the differential diagnosis from SS. There is no satisfactory differential diagnostic marker because S-100, epithelial markers, TLE1 and SOX 10 could be positive in both tumours [18]. A definitive diagnosis may only be made based on the presence of (X; 18) (p11, q11) chromosomal translocations, the cytogenetic hallmark of SS. More than 90 % of SS present this translocation, which gives rise to a fusion gene, SS18 (SYT)-SSX (SSX1 or SSX2, or both) [5–17], whereas MPNST and carcinosarcoma lack SS18-SSX fusions. The SS18 rearrangement was also observed in this case, and MPNST and metastatic carcinosarcoma were completely ruled out. The tumour readily involves the pericardium, which indicates a poor prognosis. Surgical resection was essential, although local recurrence and distant metastasis occur often post-operatively. Chemotherapy based on doxorubicin and ifosfamide has been used most commonly. The median overall survival was approximately 24 months in 36 patients in the review by Wang *et al.* [5]. This patient was cleared for chemotherapy and we intend to follow-up.

**Table 2:** Main points of differential diagnosis

Diseases	Medical history	Morphology	Immunohistochemistry						FISH (SS18)
			CK	S-100	SMA	SOX10	TLE1	Calretinin	
Primary cacdiac biphasic SS	No	biphasic	+	+/-	-	-/+	+	-	Yes
Metastatic carcinosarcoma	Yes	biphasic	+	-	+/-	-	-	-	No
Metastatic myoepithelial carcinoma	Yes	biphasic	+	+	+	-	-	-	No
Mesothelioma	No	biphasic	+	-	-	-	-	+	No
MPNST with epithelial differentiation	No	biphasic	+	+	-	+	+/-	-	No

In summary, this report describes a case of right heart biphasic synovial sarcoma, an extremely rare primary cardiac tumour that was difficult to differentiate diagnostically from metastatic tumours, particularly carcinosarcoma, by frozen and paraffin sections. Although the tumour was resected cleanly, the prognosis is unclear.

## Consent

Written informed consent was obtained from the patient for publication of this case report and any accompanying images. A copy of the written consent is available for review by the Editor-in-chief of this journal.

## Abbreviations

CT: Computed tomography
FISH: Fluorescence in situ hybridization
PCSS: Primary cardiac synovial sarcoma
MPNST: Malignant peripheral nerve sheath tumor
SS: Synovial sarcoma
